# A phase 1, randomized, open-label, single-dose study to assess the relative bioavailability of a subcutaneous dose of FKB327 when administered using a prefilled syringe, a prefilled auto-injector, or a vial with disposable syringe in healthy subjects

**DOI:** 10.1186/s40360-019-0376-9

**Published:** 2019-12-30

**Authors:** Jim Bush, Kazuki Kawakami, Rafael Muniz

**Affiliations:** 1 Executive Medical Director, Global Head, Clinical Pharmacology Physicians Covance Clinical Research Unit Limited, Springfield House, Hyde Street, Leeds, LS2 9LH UK; 2 Manager, Medical Document Group, Kyowa Hakko Kirin Co., Ltd Ohtemachi Financial City Grand Cube, 1-9-2 Ohtemachi, Chiyoda-ku, Tokyo, 100-0004 Japan; 3 Mylan Inc., 1000 Mylan Blvd. Canonsburg, Canonsburg, PA 15317 USA

**Keywords:** Adalimumab, Biosimilar, Immunogenicity, Pharmacokinetics, Safety

## Abstract

**Background/objective:**

FKB327 is a biosimilar of the adalimumab reference product (RP). The primary objective was to assess the relative bioavailability of FKB327 after a single subcutaneous (SC) dose via prefilled syringe (PFS), auto-injector (AI), or vial with a disposable syringe (vial), in healthy subjects.

**Methods:**

This randomized, open-label, parallel-group, single SC-dose study was conducted in 195 healthy male and female subjects who were randomized 1:1:1 to receive FKB327 40 mg via PFS, AI, or vial. The primary pharmacokinetic (PK) parameters, areas under the serum concentration-time curve to the last detectable value (AUC_0-t_) and extrapolated to infinity (AUC_0-∞_), and maximum concentration (C_max_), were compared. Relative bioavailability was established if the ratio of geometric least squares (LS) means of the test product was within the predefined bioequivalence (BE) range of 0.80 to 1.25 of the RP for each comparison. Safety and immunogenicity were assessed.

**Results:**

The mean serum FKB327 concentration-time profiles appeared similar across all 3 presentations. AUC_0-t_, AUC_0-∞_, and C_max_ were within the predefined BE range for PFS compared with vial, suggesting comparable bioavailability. AUC_0-∞_ and C_max_ of AI compared with vial and PFS were fully contained within BE range, although the upper limit of 90% confidence intervals of the geometric LS means ratios for AUC_0-t_ was slightly high. Treatment-emergent adverse events in all 3 groups were mild, with no new safety concern with FKB327 identified. Similar immunogenicity was observed among administrations.

**Conclusion:**

Among all 3 delivery methods, PK characteristics, safety profiles, and immunogenicity were similar.

**Trial registration:**

EU Clinical Trials Registry EudraCTN2014–004469-26, registered October 14, 2014.

## Background

Adalimumab is a recombinant, human immunoglobulin G1 monoclonal antibody specific for human tumor necrosis factor–alpha (TNF-α) that was originally approved for the treatment of rheumatoid arthritis in the United States in December 2002 and in the European Union in September 2003, and is marketed under the brand name Humira® [1, 2]. Humira was subsequently approved in the United States for juvenile idiopathic arthritis, psoriatic arthritis, ankylosing arthritis, Crohn’s disease, and plaque psoriasis [1]. In the European Union, Humira was approved for adult and pediatric Crohn’s disease, juvenile idiopathic arthritis, adult and pediatric plaque psoriasis, and adult and pediatric uveitis [2]. Analysis of clinical trial safety data and postmarketing surveillance data demonstrates that long-term treatment with adalimumab is generally safe and well-tolerated [3].

Patent restrictions for adalimumab have begun to expire. FKB327 is a biosimilar of Humira (adalimumab) [4]. Biosimilar agents are not generic forms of reference biological products. Unlike generic drugs, which are identical copies of the reference drug with the same pharmacologic effects and safety profile, biosimilars are products highly similar to the reference biologic agent but not identical [5]. Thus, whereas generic drugs can be approved on the basis of bioequivalence (BE) to the reference drug, the same is not true for the approval of biosimilars [6]. Regulatory considerations for biosimilars are continuing to evolve in the United States and European Union as a result of the distinct challenges of manufacturing biosimilars [7]. The US Food and Drug Administration (FDA) and European Medicines Agency (EMA) have both developed guidance regarding the approval of biosimilars [7]. The EMA guidance details product-specific approval pathways based on biological classification. In contrast, the FDA currently utilizes a risk-based, case-by-case, totality-of-evidence approach. To receive approval, a potential biosimilar must demonstrate similarity to the reference biologic via a stepwise approach that includes both structural and functional studies, nonclinical evaluation of pharmacokinetics (PK) and toxicity, and clinical assessment of PK, efficacy, and safety, including immunogenicity [6]. FKB327 was approved by the EMA in September 2018 and subsequently launched. FKB327 has not yet received FDA approval.

A recent randomized, double-blind, parallel-group study in healthy volunteers compared the PK, safety, tolerability, and immunogenicity of FKB327 with those of EU-approved Humira and US-licensed Humira [4]. Results from this study showed FKB327 to be well tolerated, with a safety profile similar to both Humira formulations. In addition, the PK of FKB327 were demonstrated to be similar to EU-Humira and US-Humira. Finally, the prevalence of antidrug antibodies (ADAs) and the elimination half-life of adalimumab were similar following administration of all 3 drugs.

The primary objective of this study was to assess the relative bioavailability of a subcutaneous (SC) dose of FKB327 administered via a prefilled syringe (PFS), a prefilled autoinjector (AI), or a vial with a disposable syringe (vial). Secondary objectives were to compare the safety of FKB327 across the 3 methods of administration and to evaluate the effect of body weight and injection site on the PK of FKB327.

## Methods

This study was conducted at the Covance Clinical Research Unit (CRU) in Leeds, United Kingdom. The study was not initiated until after the Medicines and Healthcare products Regulatory Agency had issued a Clinical Trial Authorisation, and the informed consent form (ICF) and protocol were reviewed and approved by the ethics committee (National Research Ethics Service Committee North East – York).

### Subjects

Subjects were healthy males and females of any ethnic origin, aged 18 to 64 years, with a body mass index (BMI) of 18.0 kg/m^2^ to 30.0 kg/m^2^. Subjects were excluded if they had previously received adalimumab. All 195 subjects enrolled in the study provided written consent after they were informed about the study and potential risks. Potential subjects were deemed healthy following medical history and an examination that included a tuberculosis test, vital signs, electrocardiography (ECG), and routine laboratory safety tests. All subjects were tested for drugs of abuse, and female subjects were tested for pregnancy. Male and female subjects of childbearing potential agreed to practice birth control from the time of dose administration until 5 months afterward. Strenuous exercise, alcohol, caffeine, and concomitant medication were restricted during the study and subjects were instructed not to smoke for 1 h prior to each blood pressure and pulse measurement.

### Design

This was a randomized, open-label, parallel-group study of a single SC 40-mg in 0.8 mL dose of FKB327 in healthy subjects to compare the relative bioavailability (assessed by PK parameters), safety, and immunogenicity of FKB327 administered via PFS, AI, or vial. A parallel-group study design was chosen, because adalimumab has a long elimination half-life and a crossover design would substantially increase the duration of the study. Furthermore, a crossover design was not considered appropriate to confirm PK characteristics in the present study, because in a previous phase 1 study comparing the PK characteristics of FKB327, EU-Humira, and US-Humira, ADAs were detected in approximately 70% of subjects across all treatment groups [4]. Subjects were assigned in a 1:1:1 ratio to receive 40 mg of FKB327 administered with a PFS, AI, or vial. Subjects were further randomized to treatment groups by weight strata (50–75 kg and > 75–100 kg) and injection-site strata (abdomen and thigh). Subjects were randomized to allocate approximately equal numbers from each stratum across treatment groups.

Subjects were screened within 28 days of dosing and were admitted to the CRU 1 day prior to dosing. Participants resided at the CRU until 24 h postdose. Subjects then returned for outpatient visits on days 3, 4, 5, 6, 7, 8, 9, 16, 23, 30, 37, 44, 51, and 65. Poststudy assessments were performed at the final outpatient visit.

### Assessments

Blood samples for serum drug concentration were taken prior to dosing and at 4, 24, 48, 72, 96, 120, 144, 168, 192, 360, 528, 696, 864, 1032, 1200, and 1536 h. Serum concentrations of FKB327 were determined using a validated immunoassay on an electrochemiluminescent (ECL) detection platform with a 96-well Meso Scale Discovery (MSD) bare (high-bind) plate, which was coated with TNF-α. A 100-ng/mL lower limit of reliable quantification was used.

Safety assessments were performed after a subject signed the ICF and then routinely up to and including the subjects’ final visit. Adverse events (AEs) were monitored during the course of the study through observation and open-ended questioning. Any reported AEs were recorded, along with any subsequent action, if required. Participants underwent assessments that included blood pressure, pulse, temperature, respiratory rate, physical examination, laboratory safety tests of blood and urine, and 12-lead ECG.

Blood samples to assess immunogenicity via the presence of ADAs were taken prior to dosing and at 360, 696, and 1536 h. The presence of ADAs was determined by a validated immunoassay on an ECL detection platform with MSD plates to detect against FKB327. Samples with percent inhibition values greater than a certain cut point were confirmed positive for the presence of adalimumab antibodies.

### Sample size and statistical methods

Sample size calculations were performed using nQuery Advisor 7.0, assuming the PK parameters, area under the serum concentration-time curve from time 0 to time t (AUC_0-t_), area under the serum concentration-time curve from time 0 to infinity (AUC_0-∞_), and maximum serum drug concentration (C_max_), were the primary end points. The intersubject variability was assumed to be 40%, and considering 80% statistical power and early-withdrawal subjects, a total of 189 subjects were expected to be enrolled for the 90% confidence interval (CI) for the ratio of geometric means of each comparison (PFS/vial, AI/vial, and AI/PFS) to fall within the acceptance criteria of 0.80 to 1.25. Because there were 3 treatment comparisons, overall power was lower than 80%; however, it was not significantly so, as the 3 tests for bioavailability are highly correlated.

Formal statistical analysis for relative bioavailability was performed on the PK data to test whether the main parameters met the predefined criteria, which was set with reference to a standard BE study [8, 9]. In the assessment of relative bioavailability, 3 comparisons were made (test product:reference product)—FKB327 PFS:FKB327 vial, FKB327 AI:FKB327 vial, and FKB327 AI:FKB327 PFS. The primary hypothesis was that FKB327 delivered via a vial, PFS, or AI would result in similar bioavailability based on the BE analysis of the geometric least squares (LS) means of AUC_0-t_, AUC_0-∞_, and C_max_. The secondary hypothesis was that FKB327 delivered via a vial, PFS, or AI would result in similar bioavailability, based on the same analysis as the primary hypothesis for the geometric LS means of elimination half-life (t_1/2_) and maximum concentration (t_max_). For each comparison, the test product was considered to demonstrate BE to the reference product (RP) if the ratio of the geometric LS means for the test product relative to the RP were contained within the 90% CI of 0.80 to 1.25.

The PK parameters of AUC_0-t_, AUC_0-∞_, C_max_, and t_1/2_ were log transformed prior to analysis and were analyzed using a fixed-effects analysis of variance (ANOVA). The model included treatment as a fixed effect. For these parameters, LS means were calculated for the test product and RP. Mean differences between the test product and RP were calculated. The residual variance from the model was used to calculate 90% CI for the difference between the test product and RP. These values were back-transformed to give geometric LS means, a point estimate, and 90% CI for the ratio of the test product relative to the RP. This procedure is equivalent to Schuirmann’s two one-sided tests at the 0.05 level of significance.

The PK parameter of t_max_ was analyzed nonparametrically using the Wilcoxon rank-sum test, with the median of the difference of t_max_ between treatments and the approximate 90% CIs for the difference presented. A secondary analysis was conducted to investigate the effect of adding body weight and injection-site strata into the ANOVA model, individually and in combination, as fixed effects. All remaining data were summarized using descriptive statistics. The analysis was performed using SAS 9.1 (SAS Institute; Cary, NC).

## Results

### Subjects

A total of 195 subjects were randomized in the study: 66 to FKB327 vial, 63 to FKB327 PFS, and 66 to FKB327 AI. Baseline characteristics are shown in Table 1. Subjects were comprised of 145 males and 50 females, with the majority of subjects being white. Demographic characteristics were generally similar among the 3 groups, with the exception of race; there were more black subjects in the AI group (13.6%) than in the vial (3.0%) and PFS (1.6%) groups. For the weight strata, 95 subjects were in the 50- to 75-kg body weight stratum and 100 were in the > 75- to 100-kg body weight stratum.

**Table 1:** Baseline Characteristics

	FKB327 vial *n* = 66	FKB327 PFS *n* = 63	FKB327 AI *n* = 66	Total *n* = 195
Age (years)				
Mean (SD)	38 (13.6)	40 (12.9)	37 (12.5)	38 (13.0)
Sex, n (%)				
Male	50 (75.8)	45 (71.4)	50 (75.8)	145 (74.4)
Female	16 (24.2)	18 (28.6)	16 (24.2)	50 (25.6)
Race, n (%)				
Asian	2 (3.0)	4 (6.3)	3 (4.5)	9 (4.6)
Black	2 (3.0)	1 (1.6)	9 (13.6)	12 (6.2)
White	62 (93.9)	58 (92.1)	53 (80.3)	173 (88.7)
Other	–	–	1 (1.5)	1 (0.5)
Weight (kg)				
Mean (SD)	76.2 (10.37)	75.2 (10.98)	75.5 (11.56)	75.6 (10.93)
BMI (kg/m^2^)				
Mean (SD)	25.2 (3.06)	25.2 (2.62)	24.8 (2.85)	25.1 (2.84)

*AI* indicates prefilled autoinjector; *BMI* body mass index; *PFS* prefilled syringe; *SD* standard deviation; vial, vial with disposable syringe

### Pharmacokinetics

**Fig. 1:**
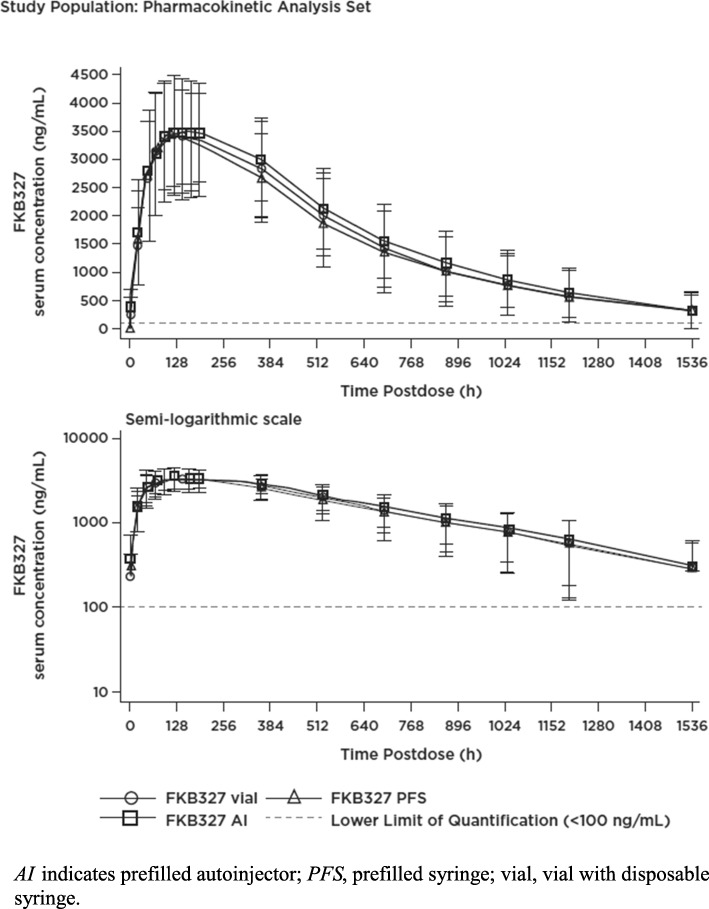
Mean Serum Concentration-Time Profiles of Adalimumab Following Subcutaneous Dose of FKB327

A total of 194 subjects were included in the PK analysis. In the AI treatment group, 1 subject was excluded because of receiving only a partial dose of the study drug. A summary of the PK parameters for each group is provided in Table 2. Mean serum concentration-time profiles of FKB327 were similar following a single SC 40-mg dose of FKB327 administered via vial, PFS, and AI (Fig. 1). FKB327 was absorbed slowly across all 3 methods of administration, with a median t_max_ of 120.03 h, 120.0 h, and 144.0 h in the vial, PFS, and AI groups, respectively.

**Table 2:** Pharmacokinetic Parameters

Parameter	FKB327 vial *n* = 65	FKB327 PFS *n* = 63	FKB327 AI *n* = 65
Primary			
AUC_0-t_ (h*ng/mL)	2,150,000 (54.7)	2,140,000 (43.0)	2,380,000 (36.5)
AUC_0-∞_ (h*ng/mL)	2,380,000 (39.1)^c^	2,300,000 (39.5)^b^	2,460,000 (38.7)^c^
C_max_ (ng/mL)	3450 (31.4)^a^	3450 (30.1)^a^	3590 (27.9)^a^
Secondary			
AUC_0-360h_ (h*ng/mL)	999,000 (41.3)	998,000 (32.4)	1,080,000 (27.4)
t_max_ (h)	120 (72.0, 363)	120 (48.0, 363)	144 (48.0, 363)
t_1/2_ (h)	305 (47.9)^c^	307 (49.7)^b^	306 (42.1)^c^
k_el_ (h)	0.00227 (47.9)^c^	0.00225 (49.7)^b^	0.00226 (42.1)^c^

^a^median (range); ^b^*n* = 56; ^c^*n* = 60

*AI* indicates prefilled autoinjector; *AUC*_*0-t*_ area under the serum concentration-time curve to the last detectable value; *AUC*_0-∞_ area under the serum concentration-time curve extrapolated to infinity; *AUC*_*0-360h*_ area under the serum concentration-time curve from zero to 360 h; *C*_*max*_ peak serum concentration; *k*_*el*_ elimination rate constant; *PFS* prefilled syringe; *t*_*max*_ maximum concentration; *t*_*1/2*_ elimination half-life; vial, vial with disposable syringe

A summary of the relative bioavailability analysis is shown in Table 3. Regarding the primary hypothesis, all primary PK parameters (AUC_0-t_, AUC_0-∞_, and C_max_) were contained within the predefined BE range for PFS compared with vial, suggesting comparable bioavailability. For the AI/vial and AI/PFS comparisons, the 90% CIs were fully contained within the BE range for AUC_0-∞_ and C_max_; however, the upper limits for AUC_0-t_ were slightly outside the BE limit of 0.80 to 1.25 (1.11 [0.976, 1.254] and 1.11 [0.981, 1.26], respectively). The secondary PK parameter of t_1/2_ was contained within the range for all 3 comparisons, as the 90% CIs for t_1/2_ were fully contained within the BE range.

**Table 3:** Summary of ANOVA for Pharmacokinetic Parameters

Parameter	Ratio of Geometric LS Means (90% CI)		
	FKB327 PFS/FKB327 vial	FKB327 AI/FKB327 vial	FKB327 AI/FKB327 PFS
Primary			
AUC_0-t_ (h*ng/mL)	0.994 (0.877, 1.13)	1.11 (0.976, 1.254)	1.11 (0.981, 1.26)
AUC_0-∞_ (h*ng/mL)	0.967 (0.861, 1.09)	1.04 (0.925, 1.16)	1.07 (0.954, 1.20)
C_max_ (ng/mL)	1.00 (0.918, 1.09)	1.04 (0.957, 1.13)	1.04 (0.957, 1.13)
Secondary			
t_1/2_ (h)	1.01 (0.880, 1.16)	1.00 (0.877, 1.15)	0.995 (0.868, 1.14)
t_max_ (h)	0 (−24.0, 0)	0 (−24.0, 23.9)	0 (0, 24.0)

*AI* indicates prefilled autoinjector; *ANOVA* analysis of variance; *AUC*_*0-t*_ area under the serum concentration-time curve to the last detectable value; *AUC*_0-∞_ area under the serum concentration-time curve extrapolated to infinity; *CI* confidence interval; *C*_*max*_, peak serum concentration; *LS* least squares; *PFS* prefilled syringe; *t*_*max*_ maximum concentration; *t*_*1/2*_ elimination half-life; vial, vial with disposable syringe

The secondary analysis of PK parameters by body weight showed a tendency for greater exposure in the 50- to 75-kg group compared with the > 75- to 100-kg group. In the 50- to 75-kg group, the geometric LS mean for AUC_0-t_ was 2,370,000, 2,470,000, and 2,790,000 h*ng/mL, and the geometric LS mean for C_max_ was 3770, 4010, and 4110 ng/mL for the vial, PFS, and AI groups, respectively. In the > 75- to 100-kg group, the geometric LS mean for AUC_0-t_ was 1,960,000, 1,860,000, and 2,040,000 h*ng/mL and the geometric LS mean for C_max_ was 3180, 2980, and 3150 ng/mL for the vial, PFS, and AI groups, respectively.

The secondary analysis of PK parameters by injection site showed a tendency for lower exposure in the abdominal group than in the thigh group. In the abdominal group, the geometric LS mean for AUC_0-t_ was 1,890,000, 202,000, and 2,350,000 h*ng/mL, and the geometric LS mean for C_max_ was 3100, 3150, and 3450 ng/mL for the vial, PFS, and AI groups, respectively. In the thigh group, the geometric LS mean for AUC_0-t_ was 2,450,000, 2,270,000, and 2,410,000 h*ng/mL and the geometric LS mean for C_max_ was 3860, 3790, and 3730 ng/mL for the vial, PFS, and AI groups, respectively.

When body weight and injection-site strata (individually and in combination) were added as fixed effects into the analysis model, all primary PK parameters (AUC_0-t_, AUC_0-∞_, and C_max_) were contained within the predefined BE range for PFS and AI compared with vial. In addition, AUC_0-∞_ and C_max_ were within the range for the comparison between PFS and AI. The upper limit of 90% CIs of the geometric LS means ratios for AUC_0-t_ were slightly outside the predefined limit (1.11 [0.987, 1.26], 1.11 [0.980, 1.26], and 1.11 [0.986, 1.251] for body weight strata, injection-site strata, and body weight and injection-site strata combined, respectively).

### Immunogenicity

The frequency of ADAs is shown in Table 4. The percentage of subjects with positive ADA status at predose was disproportionate, with 21.2, 19.0, and 10.6% of subjects showing positive ADA activity in the vial, PFS, and AI groups, respectively. At hour 360, a higher percentage of subjects in the vial group showed positive ADA activity (78.8%) compared with the PFS (54.0%) and AI groups (62.1%). The proportion of subjects with positive ADA activity at subsequent time points was nearly equal. At hour 1536, the percentage of subjects with positive ADA activity was 100% for the vial and PFS groups, and 98.5% for the AI group.

**Table 4:** Frequency of Antidrug Antibody Activity

Time point (h)	FKB327 vial *n* = 66 n (%)	FKB327 PFS *n* = 63 n (%)		FKB327 AI *n* = 66 n (%)		
	Negative	Positive	Negative	Positive	Negative	Positive
Predose	52 (78.8)	14 (21.2)	51 (81.0)	12 (19.0)	59 (89.4)	7 (10.6)
360	13 (19.7)	52 (78.8)	29 (46.0)	34 (54.0)	25 (37.9)	41 (62.1)
696	4 (6.1)	61 (92.4)	4 (6.3)	59 (93.7)	4 (6.1)	62 (93.9)
1536	0	66 (100)^a^	0	63 (100)	1 (1.5)	65 (98.5)

^a^Includes 1 early-withdrawal subject

*AI* indicates prefilled autoinjector; *PFS* prefilled syringe; vial, vial with disposable syringe

### Safety

The incidence of treatment-emergent adverse events (TEAEs) reported by ≥5% of subjects in any treatment group is shown in Table 5. A total of 125 subjects (64.1%) experienced at ≥1 AEs. All TEAEs were considered mild or moderate, with the clear majority (94.6%) considered mild. The most commonly reported TEAE for all 3 groups was nasopharyngitis, which occurred in 21.2, 23.8, and 30.3% of subjects in the vial, PFS, and AI groups, respectively. The second most commonly reported TEAE was headache, which occurred in 10.6 and 14.3% of subjects in the vial and PFS groups, respectively, compared with 3.0% in AI group.

**Table 5:** Treatment-Emergent Adverse Events Reported by ≥5% of Subjects in Any Treatment Group

	FKB327 vial *n* = 66 n (%)	FKB327 PFS *n* = 63 n (%)	FKB327 AI *n* = 66 n (%)	Total *n* = 195 n (%)
Nasopharyngitis	14 (21.2)	15 (23.8)	20 (30.3)	49 (25.1)
Headache	7 (10.6)	9 (14.3)	2 (3.0)	18 (9.2)
Cough	5 (7.6)	1 (1.6)	3 (4.5)	9 (4.6)
Oropharyngeal pain	4 (6.1)	3 (4.8)	1 (1.5)	8 (4.1)
Injection-site rash	0	0	4 (6.1)	4 (2.1)

*AI* indicates prefilled autoinjector; *PFS* prefilled syringe; vial, vial with disposable syringe

Of all the TEAEs, 53.8% were deemed possibly related to the study drug and 3.6% were deemed related to the study drug, whereas 3.1% were considered possibly related to the study device. There were no serious AEs and no discontinuations due to TEAEs. The overall incidence of TEAEs was similar in the vial and PFS groups (60.6 and 58.7%, respectively), and slightly higher in the AI group (72.7%). The higher TEAE rate in the AI group was accounted for by a greater incidence of nasopharyngitis, injection-site rash, vessel puncture–site pain, and vessel puncture–site bruise.

A total of 4 subjects had laboratory results of potential clinical importance. Increases in transaminases deemed to be clinically significant were observed in 2 subjects in the AI group, and were reported as TEAEs. An increase in creatine kinase was also noted for 1 of these subjects and was reported as a TEAE. Increases in leukocytes and neutrophils deemed clinically significant were observed in 2 subjects and were reported as a TEAE for 1 of the subjects. A decrease in neutrophils considered clinically significant was noted for 1 subject and was reported as a TEAE. There were no clinically meaningful findings in vital signs or 12-lead ECGs for any subjects during the study.

## Discussion

In this study, we sought to confirm the similar bioavailability of FKB327 whether delivered by vial, PFS, or AI, as assessed using a typical BE range across the PK parameters of AUC_0-t_, AUC_0-∞_, and C_max_. All primary parameters (AUC_0-t_, AUC0_-∞_, and C_max_) were contained within the predefined BE range for PFS compared with vial, suggesting comparable bioavailability, and AUC_0-∞_ and C_max_ were contained within the BE range in the AI/vial and AI/PFS comparisons. For the AI/vial and AI/PFS comparisons, the 90% CIs for AUC_0-t_ were slightly outside the predefined BE limit. No statistically significant difference was found in t_1/2_ among the 3 methods of administration.

Analysis of body weight and injection-site strata showed that exposure for the 50- to 75-kg group was slightly higher than in the > 75- to 100-kg group as expected, and the thigh group showed a tendency for greater exposure than the abdomen group. Based on the apparent effect of body weight as a categorical fixed effect, 2 post hoc analyses were performed to explore the effect of body weight and BMI on the BE of the primary PK parameters. First, the primary statistical analysis was repeated with the addition of body weight as a covariate to the statistical model. Second, the primary statistical analysis was repeated with the addition of BMI as a covariate to the statistical model. When body weight was included as a covariate, the primary PK parameters of AUC_0-∞_ and C_max_ were within the BE range for PFS and AI compared with vial; however, the upper limit of the 90% CIs of the geometric LS means ratios for AUC_0-t_ was slightly outside the BE range of 0.80 to 1.25. When BMI was included as a covariate, all primary PK parameters (AUC_0-t_, AUC_0-∞_, and C_max_) were contained within the predefined BE range for all 3 methods of administration.

The proportion of subjects with positive ADA activity at baseline was lower for the AI group (10.6%) than for the vial (21.2%) and PFS (19.0%) groups, suggesting possible slight differences in immunogenicity conditions in the AI group. Due to the imbalance in the ADA-positive ratio by confirmatory assay at baseline, an additional post hoc statistical analysis, adjusted by baseline (predose) ADA status, was performed. When adding baseline confirmatory ADA status as a fixed effect to the analysis model, primary PK parameters of AUC_0-t_, AUC_0-∞_, and C_max_ were fully contained within the predefined BE range for all 3 methods of administration.

Single SC doses of 40-mg FKB327 were well-tolerated by healthy male and female subjects whether delivered by a vial, PFS, or AI. The rate of TEAEs was slightly higher for the AI group, largely due to higher incidence of nasopharyngitis, injection-site rash, vessel puncture–site pain, and vessel puncture–site bruising reported in this group. The majority of TEAEs in all 3 groups were of mild severity (94.6%). No severe TEAEs were reported and no subjects discontinued because of a TEAE. Furthermore, no safety concerns were found based on clinical laboratory evaluations, vital signs, and 12-lead ECGs.

The results shown here advance findings from a previous study of FKB327 in healthy volunteers [4]. Puri and colleagues reported that FKB327 was well-tolerated, with a safety profile similar to Humira. Furthermore, the PK characteristics of FKB327 were similar to both US and EU formulations of Humira.

## Conclusions

A single SC 40-mg dose of FKB327 was well-tolerated in healthy subjects whether delivered by a vial, PFS, or AI. In addition, the relative bioavailability of FKB327 delivered across all 3 methods of administration was highly similar based on the analysis of the PK parameters. The ADA activity and t_1/2_ of adalimumab were also similar for all 3 methods of administration. The results from this study suggest that the delivery of FKB327 with a vial, PFS, or AI can be used interchangeably in clinical practice.

## Data Availability

The datasets generated during and/or analyzed during the current study are available from the corresponding author on reasonable request.

## Abbreviations

ADA: Antidrug antibody
AE: Adverse event
AI: Autoinjector
ANOVA: Analysis of variance
AUC_0-∞_: Area under the serum concentration-time curve from time 0 to infinity
AUC_0-t_: Area under the serum concentration-time curve from time 0 to time t
BE: Bioequivalence
BMI: Body mass index
CI: Confidence interval
C_max_: Maximum serum drug concentration
CRU: Clinical Research Unit
ECG: Electrocardiography
ECL: Electrochemiluminescent
EMA: European Medicines Agency
FDA: Food and Drug Administration
ICF: Informed consent form
LS: Least squares
MSD: Meso Scale Discovery
PFS: Prefilled syringe
PK: Pharmacokinetics
RP: Reference product
SC: Subcutaneous
t_1/2_: Elimination half-life
TEAE: Treatment-emergent adverse event
t_max_: Maximum concentration
TNF-α: Tumor necrosis factor–alpha
